# Setting Pivot Teeth

**Published:** 1858-11

**Authors:** J. D. Wingate

**Affiliations:** Bellefonte, Pa.


For the Dental Reporter.

### SETTING PIVOT TEETH.

In the practice of Dentistry we find many Pivot Teeth, after having been worn for some time, become exceedingly offensive; owing often to imperfect adaptation of the tooth to the root. In many cases of resetting we find the root so funnelled out that a good fit can not be had without much trouble and expense. It has for some time been the writer's practice to prepare gutta percha, by mixing with it as much pulverized silex as it will hold, without becoming brittle, (mix by heat over a small lamp on silver plate.) This Mixture becomes quite hard and has, after a year's wearing, been found as good as when put in the root.

After having adapted the tooth to the root and the pivot fitted up and in the tooth, I cut off a little of the prepared gutta percha and put it on the pivot close to the tooth, hold it over a small alchohol lamp far enough from the blaze merely to become softened, then press it around the pivot; then I hold it over the lamp again, long enough to become quite hot, but not hot enough to burn or even discolor the gutta percha; it must then be put into its place immediately, the root having been previously dried out. This will make a good joint and a very substantial tooth.

In resetting teeth, the root when funnelled out, may, by a middling fit set with gutta percha, be made to hold a tooth quite firmly.

On *pressing occasions*, when a plate tooth was not desired, I have delighted patients by resetting pivot teeth that had been out several years; the roots having been condemned as not sufficiently strong to hold a pivot.

BELLEFONTE, PA. J. D. WINGATE.

